# Prevalence and Differences of Depression, Anxiety, and Substance Use Between Chinese College-Age Students Studying in China and America During the Coronavirus Disease 2019 Pandemic

**DOI:** 10.3389/fpsyt.2022.805120

**Published:** 2022-03-16

**Authors:** Mingsheng Li, Wangdi Sun, Ye Wang, Chang Qi

**Affiliations:** ^1^Department of Geriatric VIP No. 3 (Department of Clinical Psychology), Rehabilitation Medicine Center, Zhejiang Provincial People's Hospital, Affiliated People's Hospital, Hangzhou Medical College, Hangzhou, China; ^2^Psychology Department, Denison University, Granville, OH, United States; ^3^Psychology Department, Air Force Health Care Center for Special Services Hangzhou, Hangzhou, China

**Keywords:** anxiety, depression, substance use, college students, COVID-19

## Abstract

**Introduction:**

The coronavirus disease (COVID-19) swept the globe and harmfully influenced the mental health and behaviors of the college student population. This study aims to examine the prevalence and difference of mental health and the substance use problems of the Chinese college-age students studying in China and America (CSA) during the COVID-19 pandemic.

**Methods:**

One thousand five hundred four students participated in this study. A total of 42.12% of students are enrolled in Chinese colleges, and 57.78% of students are enrolled in American colleges, aged 17–23 years ($\bar{x}\pm s$ = 19.90 ± 1.50). Binary logistic regression and independent *t*-test were used in this study to find the predictor variables and association among mental health, substance use problems, and student population.

**Results:**

The two student groups had a statistical difference in General Anxiety Disorder-7 scale, alcohol, medicines, drinks, drugs and cigarettes (*p* < 0.01). The students suffering depression problems from the two groups have statistical significance with drinks (odds ratio = 0.89, 95% confidence interval = 0.81–0.97, *p* < 0.05; odds ratio = 1.11, 95% confidence interval = 1.04–1.19, *p* < 0.01). CSA experiencing anxiety problem had a significant association with alcohol, drinks, cigarette, and desserts (*p* < 0.05).

**Conclusion:**

This is the first cross-sectional study focusing on the comparison of the Chinese college-age students' mental health and substance use problems who are studying in China and America during the pandemic. Our study revealed severe mental health and substance use problems in both student groups, particularly in the CSA during the COVID-19 pandemic. The findings of our study also highlight the evidence to find more interventions and preventions to solve the different mental health and substance use problems for college students.

## Introduction

The outbreak of health emergencies resulting from coronavirus disease 2019 (COVID-19) not only negatively impacted the mental and physical health of the general population but also increased the incidence of psychological crises (1, 2). A growing literature revealed that many individuals were experiencing severe psychological burden during this period across countries (3–6); stressors involve social isolation, health concerns, fears of being infected, and stigma, which resulted in a series of negative emotional problems, fear, anxiety, and frustration.

Most college students are in the phase of stepping into their adulthood and are usually under tremendous pressure from academic and social lives due to most of them are living independently for the first time (7, 8). Xiang et al. found that the educational environment changes could lead them to increase stress and perform poorly, influencing their cognition and motivation and generating feelings such as fear and anxiety, especially during the pandemic. According to the previous research report, during the COVID-19 pandemic, Chinese college students' anxiety and depression levels were higher than the national norm level (9–11). The lockdown of schools caused by the COVID-19 pandemic created a significant negative impact on these students' mental health and substance use, which have entirely changed their academic and social life across the countries (3, 4, 7). Chinese domestic college students were at high risk of common mental health problems, such as depression, anxiety, and substance use problems (9, 10).

Although the Chinese college students studying in China (CSCs) were significantly impacted by the COVID-19 pandemic, the Chinese international college students in America (CSAs) have also been influenced negatively. Most educational institutions were shut down in mid-March in 2020 in the United States, and all students were forced to shift their classes into the remote model *via* Zoom and Google Meet (12), which led most of these international students to go back to China. The alternation of the teaching model and the huge time difference resulted in anxiety, and sad feelings for these students have emerged during the pandemic. Furthermore, the COVID-19 pandemic caused these students to be not able to attend commencement in person but to take a semester/year gap or transfer back to Chinese colleges. They also had to stop working on their internships and research projects (13, 14). For social lives, many racist and violent reports in the TV and news have significantly increased and escalated against Asians, including Chinese in America, because the first case was reported in China. It is reported that Chinese international students have experienced these negatively biased and perceived discriminations and anxiety during the period (15–17). A profound long-term negative influence, such as depression and anxiety levels, has changed Chinese college students' experiences in America (5). Against this background, we want to find the difference between CSC and CSA by analyzing the prevalence of mood disorders and substance use problems to provide evidence for the prevention and clinical treatment of mental health problems of the college students' population. Furthermore, considering that COVID-19 is a global challenge that impacts college students' mental health globally, it is also urgent to obtain information regarding the problems in different countries caused by the pandemic and potential factors that can influence its extent in different countries.

In addition to mental health problems, substance use problem is also becoming a severe issue among the college student population and is closely associated with mental health. The previous study reported that college students formed a large proportion of binge drinkers among young generations, and 70% of American college students were drinkers in 2007 in the United States (18, 19). At the same time, cigarettes have similar results, with a marked increase in smoking rates among people with a high level of education, college students, in China. A total of 53% of the Chinese participants in a study reported having smoked during their college (20, 21). The non-medical use of prescription drugs (NMUPD) is also a health issue among college students. It was previously reported that college students have a high prevalence of NMUPD in the United States, whereas it also concluded that NMUPD in Chinese college students appears to be common recently (22). Besides, over 90% of students reported buying coffee and energy drink almost every day (23, 24). The recent studies also indicated that the college students' substance use situation, including alcohol, cigarette, and marijuana (25–27), was more frequent and severe than before the pandemic. Thus, it is also emergent and important to examine how substance use has changed during the pandemic and how it could relate to mood disorders to give evidence in solving the future problems of mental health and substance abuse among college students.

To facilitate the development of domestic and international college students' mental health and substance use intervention programs, we need a better understanding of both the epidemiology of depressive and anxiety symptoms and substance use in CSC and CSA amid the COVID-19 pandemic. Although many researchers are investigating these issues in CSCs, the research of CSAs is limited. To better help policymakers and psychologists on this issue, this study analyzes and compares prevalence in mental health by taking the sample from CSC and CSA during the COVID-19 pandemic. The hypothesis of the present study is that both CSCs and CSAs will experience mood problems, depression, and anxiety. Based on a previous study (5), CSAs would experience more severe mood and substance abuse problems than those studying in China during the pandemic. The students in America are expected to experience more frequent alcohol and drug use problems than students in China. The findings of our study could give some evidence for establishing culturally tailored prevention intervention programs to provide a direction for solving the mental health and substance use of college students, even extend to all college students across the world to decline the risk of future public health emergencies.

## Methods

### Participants

As approved by the Denison University and Zhejiang Provincial People's Hospital's Institutional Review Board, the authors developed and released an online and anonymously survey on the official website of “Questionnaire Star,” a professional online questionnaire and evaluation platform. Participation was voluntary, and their responses to this survey would be truly anonymous, which means that it ultimately ensures that no one could ever identify their responses to who they are.

To recruit the student participants, we published an online questionnaire on the website of “Questionnaire Star” and spread it into social media. One thousand six hundred forty-five participants were collected through social media and the website of “Questionnaire Star.” Questions such as present location, study model (hybrid/in-person/remote/gap), highest education, and class year were asked before answering the survey to recruit the student participants. We excluded (1) participants who still live in America during the collecting period to make all student participants experience a similar environment, to diminish errors; (2) those whose age does not fit the undergraduate school range; (3) participants who do not fully complete the questionnaires; (4) students who are taking a semester/year gap; and (5) students who are not presently enrolled in college. As a result, 1,504 students participated in this study and were distributed to the CSC group (*n* = 635) and the CSA group (*n* = 869). There were 42.15% men and 57.85% women, and the age range is from 17 to 23 years ($\bar{x}\pm s$ = 19.90 ± 1.50).

### Measures

The online survey is divided into four main sections, and the specific items and scores are shown in Tables, including the demographic questions (age and sex), Patient Health Questionnaire-9 (PHQ-9), General Anxiety Disorder-7 scale (GAD-7), and substance use questions. The PHQ-9 is a self-rating survey of the diagnostic instrument for common mental disorders, which showed good reliability and validity in a previous study (28). The GAD-7 is a seven-item anxiety survey that had good reliability, as well as an internal, criterion, construct, current, and convergent validity (29). The substance use scale was designed by our research team and assessed the participants' frequency of substance use per week, such as alcohol (beer, whiskey, etc.), medicines (sleeping pills, dietary supplements, etc.), desserts (cake, ice cream, etc.), drinks (coffee, bubble tea, etc.), drugs (marijuana, nitrous oxide, etc.), cigarettes (tobacco, e-cigarette, etc.), and snacks (Chex Mix, pretzels, etc.). Based on these score criteria of each questionnaire, this study would assign whether the students are experiencing mental health disorders or substance use problems.

### Statistical Analyses Approach

This utilized the Statistical Package for the Social Sciences (SPSS) 25.0 to analyze the data. Demographical information and sex used the Chi-square test to find the relationship between sex and students' population. Independent *t*-tests were used to find out the difference in mental health levels and substance use situation between Chinese domestic college students and Chinese international college students. The binary logistic regression analysis was used to examine the prediction and correlation between substance use issues and mental health problems across two student populations. A two-tailed *p*-value of < 0.05 was considered significant for all tests. We used Cox and Snell *R*^2^ and Nagelkerke *R*^2^ to evaluate our model. The larger the value of *R*^2^, the better the model's goodness of fit. Both student groups showed relatively good goodness of fit.

## Results

**Figure 1:**
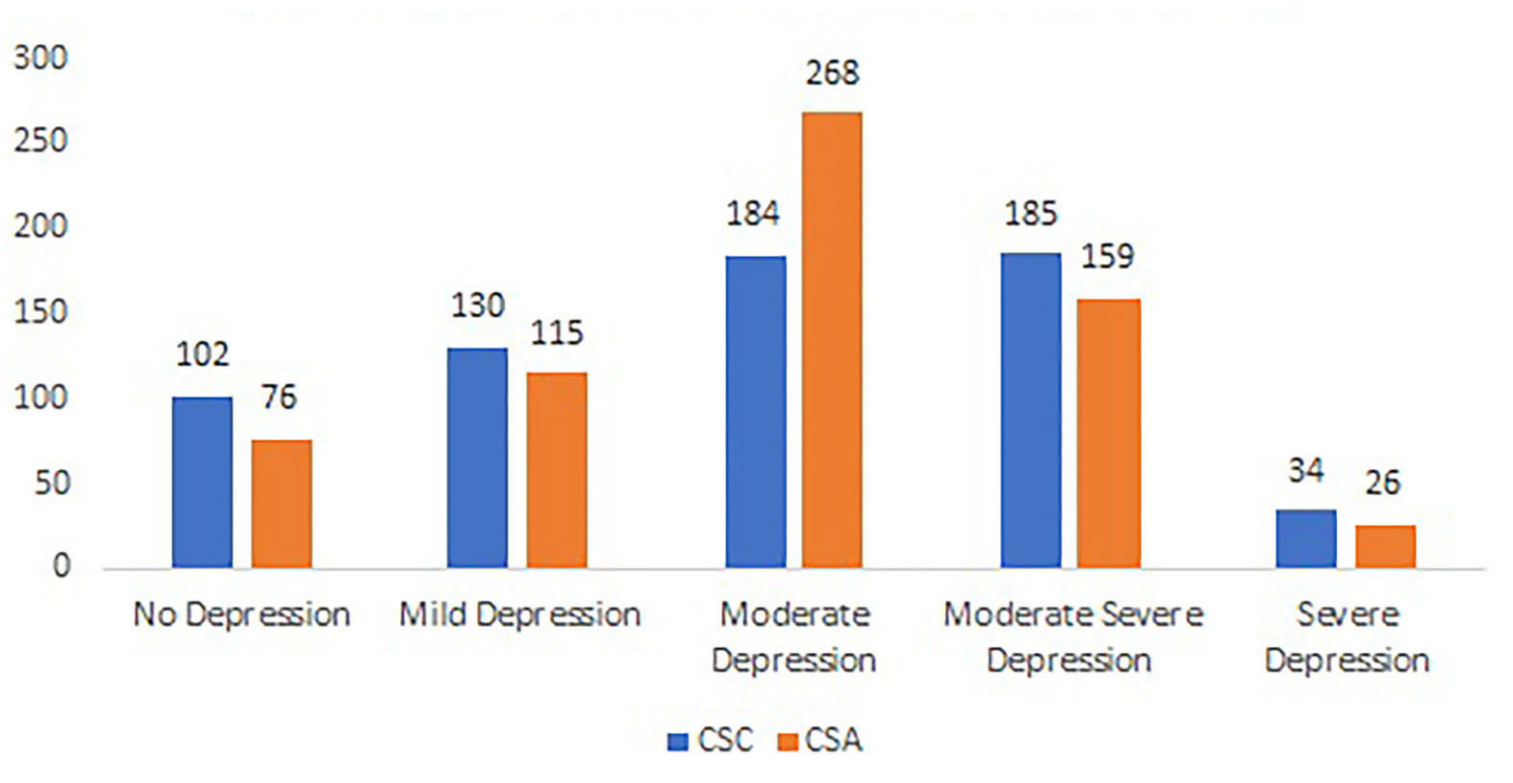
The prevalence of depression between CSC & CSA.

**Figure 2:**
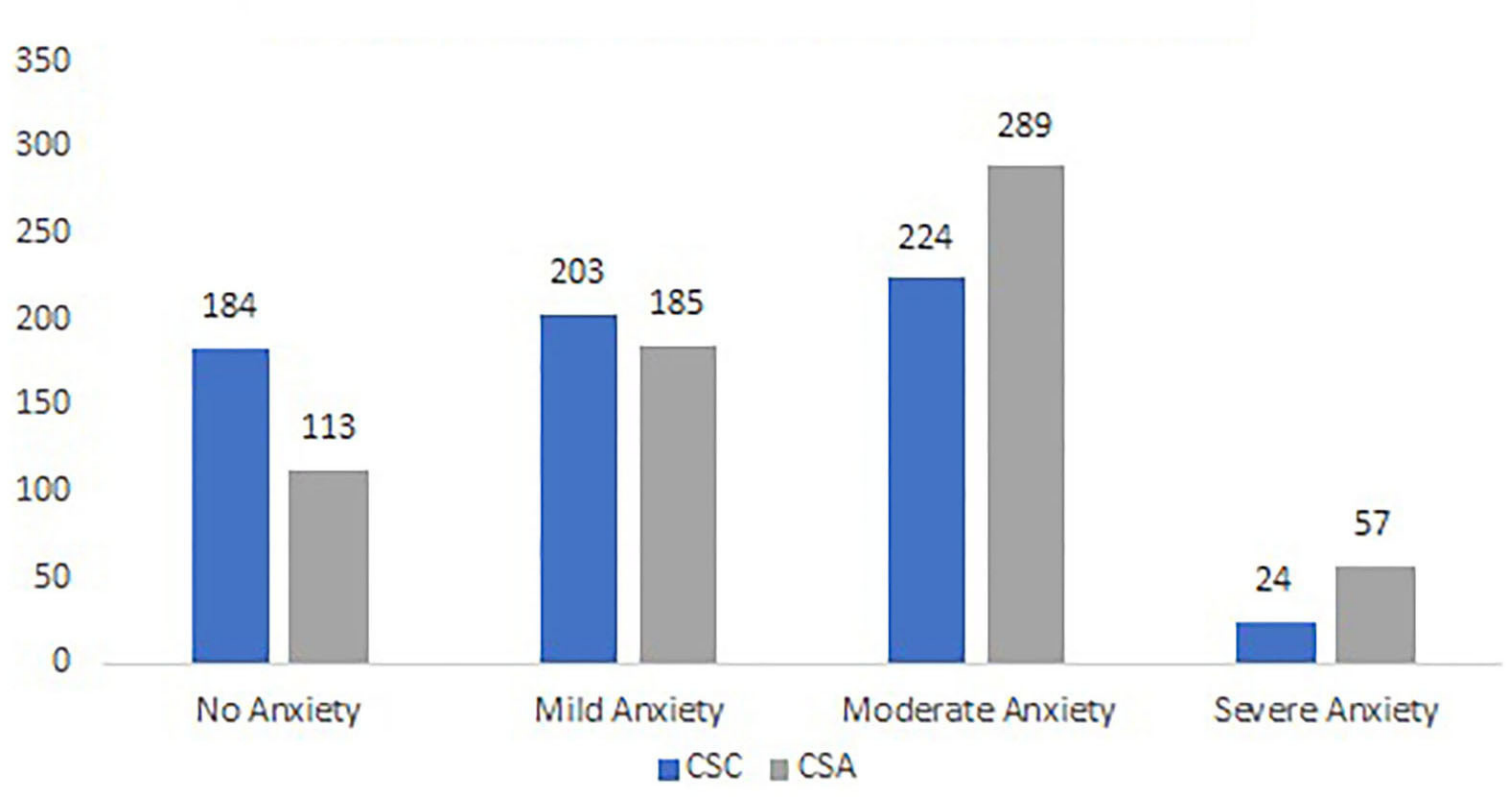
The prevalence of anxiety between CSC & CSA.

Table 1 shows the fundamental demographic characteristics and results of PHQ-9 and GAD-7 of the two student groups. A Chi-square test of independence showed no significant association between sex and student population, χ2 _(1, 1, 504)_ = 3.9, *p* > 0.05. There was no significant effect of age and PHQ-9 scores (*p* > 0.05) between the students studying in China and America. In contrast, there is a significant difference in GAD-7 mean scores (*t* = −5.46, *p* < 0.01), which indicates that the college students studying in America (*M* = 8.87, *SD* = 4.74) had a high anxiety level than in China (*M* = 7.42, *SD* = 5.52). Although there is no statistical difference for the PHQ-9 scores between the two groups, the number of CSAs (*n* = 776) who experienced depression is more than the number of CSCs (*n* = 533). Figures 1, 2 show the specific levels for depression and anxiety based on the score criteria of PHQ-9 and GAD-7. Both groups show a high rate of moderate depression (29.0 vs. 41.6%) and anxiety (35.3 vs. 44.9%). In other words, both student groups were suffering serious mental health problems impacted by the COVID-19 pandemic.

**Table 1:** Descriptive information of 1,504 college students ($\bar{x}\pm s$).

**Characteristics**	**CSC** **(***n*** = 635)**	**CSA** **(***n*** = 869)**	***χ2/t***	***p***
Gender	1.61 ± 0.49	1.56 ± 0.50	3.9	0.05
Age	19.87 ± 1.48	19.92 ± 1.48	−0.66	0.51
PHQ-9	11.63 ± 5.45	11.41 ± 5.04	0.80	0.42
GAD-7	7.42 ± 5.52	8.87 ± 4.74	−5.46	<0.01**

** *Means significant at the 0.01 level (two-tailed)*.

Table 2 provides the comparison between CSC and CSA's frequency of substance use per week by using independent group *t*-test analysis. The results show that CSC had a more severe substance use problem with alcohol (53.7 vs. 56.2%, *p* < 0.01) and drinks (77.6 vs. 61.6%, *p* < 0.01). In contrast, CSAs experienced more serious issues with medicines (41.7 vs. 57.3%, *p* < 0.01), drugs (0.9 vs. 48.3%, *p* < 0.01), and cigarettes (40 vs. 57.4%, *p* < 0.01). In other words, under the influence of the COVID-19 pandemic, both student groups experience the substance use issue, especially for CSAs.

**Table 2:** Frequency of substance use per week of students who study in China and America (*n* = 1,504).

	**CSC**		**CSA**			
	**(total** ***n*** **= 635)**		**(total** ***n*** **= 869)**		***t***	***p***
	***n***	**%**	***n***	**%**		
**Frequency of substance use per week**						
**Alcohol**					3.77	<0.01**
0 times	294	46.3	381	43.8		
1–3 times	217	34.2	432	49.7		
4–6 times	117	18.4	56	6.4		
>7 times	7	1.1	/	/		
**Medicine**					−5.42	<0.01**
0 times	370	58.3	371	42.7		
1–3 times	194	30.6	378	43.5		
4–6 times	68	10.7	88	10.1		
>7 times	3	0.5	32	3.7		
**Desserts**					0.51	0.61
0 times	149	23.5	334	38.4		
1–3 times	358	56.4	404	46.5		
4–6 times	96	15.1	52	6.0		
>7 times	32	5.0	79	9.1		
**Drinks**					4.44	<0.01**
0 times	142	22.4	334	38.4		
1–3 times	333	52.4	411	47.3		
4–6 times	118	18.6	74	8.5		
>7 times	42	6.6	50	5.8		
**Drugs**					−16.02	<0.01**
0 times	629	99.1	449	51.7		
1–3 times	1	0.2	384	44.2		
4–6 times	5	0.8	36	4.1		
>7 times	/	/	/	/		
**Cigarette**					−4.24	<0.01**
0 times	381	60.0	370	42.6		
1–3 times	168	26.5	396	45.6		
4–6 times	72	11.3	48	5.5		
>7 times	14	2.2	55	6.3		

** *Means significant at the 0.01 level (two-tailed)*.

To find the correlation among the substance use frequency, GAD-7, and PHQ-9 scores for the two groups, this study generated a binary logistic regression analysis. The results reveal in Table 3 that CSCs experiencing depression have a significant association with alcohol [OR = 0.85, 95% confidence interval (CI) = 0.77–0.95, *p* < 0.01] and drinks (OR = 0.89, 95% CI = 0.81–0.97, *p* < 0.05). The probability rates of CSCs with depression problems drinking alcohol and beverages are 0.85 and 0.89 times, respectively, higher than those without depression problems. On the other hand, CSAs experiencing depression have a significant association with drinks (OR = 1.11, 95% CI = 1.04–1.19, *p* < 0.01) and cigarettes (OR = 1.14, 95% CI = 1.06–1.22, *p* < 0.01), indicating that the probability rates of CSAs with depression of drinking beverages and smoke are 1.11 and 1.14 times, respectively, higher than those without depression problems. These students also had a significant association with desserts (OR = 0.90, 95% CI = 0.85–0.95, *p* < 0.01) and drugs (OR = 0.82, 95% CI = 0.71–0.95, *p* < 0.05), which indicates that the probability rates of these students to eat desserts and use drugs are 0.90 and 0.82 times, respectively, higher than those without depression.

**Table 3:** Binary logistic regression analysis for depression disorder for two student population.

	**CSC**							**CSA**						
	**B**	**SE**	**β**	***p***	**OR**	**95% CI**		**B**	**SE**	**β**	***p***	**OR**	**95% CI**	
						**Lower**	**Upper**						**Lower**	**Upper**
Alcohol	−0.16	0.05	8.97	<0.01**	0.85	0.77	0.95	0.10	0.05	3.63	0.06	1.11	1.00	1.23
Medicine	−0.01	0.06	0.02	0.88	0.99	0.87	1.12	−0.07	0.04	3.33	0.07	0.93	0.87	1.01
Dessert	0.04	0.06	0.46	0.50	1.04	0.93	1.16	−0.11	0.03	12.13	<0.01**	0.90	0.85	0.95
Drinks	−0.12	0.05	6.26	<0.05*	0.89	0.81	0.97	0.10	0.03	9.25	<0.01**	1.11	1.04	1.19
Drugs	−0.12	0.22	0.29	0.59	0.89	0.58	1.37	−0.20	0.07	6.97	<0.05*	0.82	0.71	0.95
Cigarette	−0.06	0.06	1.34	0.25	0.94	0.84	1.05	0.13	0.04	12.62	<0.01**	1.14	1.06	1.22

*Significant p < 0.05*

* and < 0.01**.

*CI, confidence interval; OR, odds ratio*.

Table 4 indicates that the CSA experiencing anxiety problem had a significant association with alcohol (OR = 1.15, 95% CI = 1.04–1.27, *p* < 0.05), drinks (OR = 1.11, 95% CI = 1.04–1.18, *p* < 0.01), and cigarette (OR = 1.16, 95% CI = 1.08–1.24, *p* < 0.01). These students also have a significant association with desserts (OR = 0.90, 95% CI = 0.85–0.96, *p* < 0.01). These results mean that the probability rates of CSAs with anxiety problems of drinking alcohol and beverages, eating desserts, and smoking are 1.15, 1.11, 0.90, and 1.16 times, respectively, higher than students without anxiety problems. However, there is no statistical significance for CSC. The error in the drug variable was caused by the extremum of non-drug takers in students studying in China.

**Table 4:** Binary logistic regression analysis for anxiety disorder for two student population.

	**CSC**							**CSA**						
	**B**	**SE**	**β**	***p***	**OR**	**95% CI**		**B**	**SE**	**β**	***p***	**OR**	**95% CI**	
						**Lower**	**Upper**						**Lower**	**Upper**
Alcohol	0.01	0.05	0.03	0.87	1.01	0.92	1.11	0.14	0.05	7.31	<0.05*	1.15	1.04	1.27
Medicine	0.03	0.06	0.29	0.59	1.03	0.92	1.15	0.02	0.04	0.26	0.51	1.02	0.95	1.10
Dessert	0.06	0.05	1.34	0.25	1.06	0.96	1.16	−0.10	0.03	11.03	<0.01**	0.90	0.85	0.96
Drinks	−0.06	0.04	2.01	0.16	0.95	0.87	1.02	0.10	0.03	9.79	<0.01**	1.11	1.04	1.18
Drugs	6.15	3,980.35	0.00	1.00	466.91	0.00	/	−0.11	0.07	2.10	0.15	0.90	0.78	1.04
Cigarette	0.06	0.05	1.25	0.26	1.06	0.96	1.17	0.14	0.04	16.44	<0.01**	1.16	1.08	1.24

Significant p < 0.05* and < 0.01**.

*CI, confidence interval; OR, odds ratio*.

## Discussion

This is the first cross-sectional study investigating depression, anxiety, and substance use problems among CSCs and CSAs during the COVID-19 pandemic. There are two significant findings that are consistent with the previous hypothesis. First, our results indicated that most of our participants were in a depressive and anxious mood, whereas the CSAs had a relatively higher anxiety level. Secondly, both CSCs and CSAs showed problems with the substances: CSCs had a higher tendency to drink alcohol and beverages, and CSAs were more likely to be addicted to medicine, drugs, and cigarettes. Furthermore, these two groups of students with depression problems are more likely to drink beverages.

Both groups of students showed a high prevalence of depression in the study. Compared with the 23.8% prevalence of depression among Chinese college students during the non-COVID-19 period (10), the depression rate (35.4%) of Chinese college students during the COVID-19 pandemic appeared higher, which is consistent with the previous study (8, 9, 30). Despite the difference in the number of participants for the two studies, these results indicated the elevated risk of depression in Chinese college students during the pandemic. Both student groups struggled with many issues and experienced high depression and anxiety levels influenced by the COVID-19 pandemic; however, CSAs faced more severe problems. Of course, there are some pre-differences that can potentially contribute to the differences we identified. For example, the drive for higher education is different between Chinese and American college students. CSAs may tend more to consider higher education abroad as a life-changer, as they believe that studying abroad would enable them to understand the differentiation of personal development and contribute to their improvement (31) and thus might cause them to experience more severe emotional symptoms.

Many American universities were forced to close suddenly and changed the learning model from in-person to remote, which led to discontinuing their studies, research projects, and internships. As a result, their stress level increased to a peak with various stressors (5, 12). The social life of these students has also been harmfully influenced, as they have to be separated from their friends, classmates, and professors for an extended period, which increases their loneness. The feelings of social isolation are on the rise, as they believe they may not see their friends anymore, and some cannot even say goodbye to each other (32, 33). Besides, discrimination against the Chinese during the COVID-19 pandemic may also lead to a higher level of anxiety. The news against China and Asia across the world stemmed repeatedly. For example, one of the most notable anti-Asian COVID-19 discrimination was spoken by the former President of the United States, referring to the virus as the “China virus” (15–17). As a result, the Chinese international college students were under severe negative bias and lived in fear of being discriminated against, which significantly influenced their mood and self-esteem. Furthermore, going back to China caused the time differences issue, where these students have to stay up late to take classes remotely, leading to poor physical health (9, 34). These “multiple jeopardies” overwhelmed these students and made their anxiety and stress levels rapidly rise.

Our results also showed that both student groups have substance use problems. CSCs were facing more severe alcohol and beverage issues, as the minimum age for buying alcohol is 18 years in China, so Chinese college students are more accessible to get it than students in America (16, 35). Besides, alcohol use in moderation is considered good for the health in China. Therefore, it is difficult for these Chinese college students to manage the capacity for liquor. With immature minds and scanty experiences of life, they would easily be drinking alcohol excessively when others urge them to drink at a party or social event (36). Loneliness could also lead these Chinese college students to overly drink alcohol, so they frequently use alcohol to ease their pain and alleviate their negative feelings (37–41). Other studies also indicated that loneliness was more common among people, such as healthcare workers, exposed to COVID-19, stressing the role of potential to counteract the adverse effects of higher levels of loneliness (39–41). Coffee is one of the most popular drinks among college students (42), particularly for students who have had insufficient sleep. They need energy and keep sober to reduce fatigue to listen to the class and prepare for the exams during the daytime (24, 43). Thus, these college students seek caffeine from coffee to increase energy and improve their study performance, as caffeine is the most frequently consumed central nervous system stimulant in the world (44, 45).

CSAs were experiencing more severe problems in medicines, drugs, and cigarettes. The number of CSAs may be impacted subtly by the western culture with higher drinking and smoking habits (20, 22). One of the examples of medicines in our survey is dietary supplements. The students who live on the American campus may be influenced imperceptibly by the American lifestyle and eating habits of eating dietary supplements to make a healthy and strong body (46). During the COVID-19 pandemic, taking dietary supplements every day would be effective, as everyone believes that they would be protected from the virus if they have a healthy body (47). There are several psychological and physical benefits of taking these dietary supplements: obtaining adequate amounts of nutrients and maintaining their health, losing weight, preventing diseases, and improving immunity (48–50). Furthermore, sleeping pills such as melatonin are also examples in our medicine category. College students are usually associated with sleep difficulties and insufficient sleep, where they are constantly forced to stay up late by the academic stress and assignment workload (51–53). Meanwhile, after these CSAs returned to China, they needed to stay up late to take the remote class. Therefore, these students' sleep patterns and biological clock have been disturbed. Their physical and psychological health collapsed under the pressure of stress from academic life, social activities, and internship, and this explains why they would frequently use sleep medicines to have a good sleep or immediately fall asleep.

Prior research concluded that college students are one of the groups easy to be addicted to smoking (54). Electronic cigarettes (E-cigarette) have increased in popularity among college students and have become the most common tobacco product among this generation (55). During the COVID-19 pandemic, these college students were suffering from the pressure from academic, social, and daily routines. Compared with cigarettes, the E-cigarette is a “healthy product” that could make them quit/reduce tobacco use and release stress (56, 57). Many studies have revealed that youths who use tobacco products (including e-cigarettes) tend to use cannabis than non-tobacco users (58, 59). Similar problems happen with drugs. Late adolescence and young adulthood, the college-age period, are peak developmental periods for drug involvement. Non-medical use of prescription medications is one of the common phenomena, including analgesics and analeptics, in Chinese college students (22, 60). The students who take drugs might believe that the drugs could help them get rid of low emotions and avoid annoyance easily. Another benefit of the drugs could be to medicate sleep problems. The students reported that sleep onset is more rapid after marijuana use (61, 62).

The present study also confirmed a known association between substance use and affective symptoms (depression and anxiety) in both student groups. The social mechanism might be one of the explanations. As the school and government policies established, most of the students needed to study at home. Under tremendous stress from academics, parents, and graduation, the inside feelings of loneliness and frustration of these students were exaggerated (63). A previous study found that binge eating and drinking could be one of the symptoms caused by loneliness (64), which results in loneliness being the possible reason causing these college students to use substances frequently. In addition to the substance use problem, loneliness also caused adverse effects on students' mental health, such as strong associations with suicide ideation and attempts (65) and depressive mood (63). The student population has severe mental health problems and frequent substance use issues that might be attributed to extreme loneliness.

In conclusion, the mental health and substance use problem of the Chinese student population study in China and America affected by the epidemic differs significantly, especially for CSAs. It is imperative to vigorously develop mental health services in China and promote mental health for these college students more deeply and broadly. The effective method is examining the problems in a group or sample of college students and finding out the problems, then taking measures to deal with each problem that these college students have. We recommend that professors, counselors, and administrative staff working in colleges facilitate taking necessary steps to help these students, including self-help brochures, psychoeducational studies, and individual and group counseling. Thus, these people who work in colleges should proactively reach out to all student populations, identify students who need help or are at high risk of mental health problems, and plan tailored treatment to counteract the adverse psychological effects. In addition, future tailored prevention intervention programs and more discussion are needed in China about the regulation of substances that may be beneficial to reduce the risk of substance use among Chinese college students. After the series mentioned earlier are implemented, the risk of students being infected can be reduced, at the same time, lower mental health problems. Even relevant health departments could establish a reasonable system that can be extended to decline the risk of future public health emergencies.

This study has several limitations that should be improved in future studies. First, its sample size was relatively small, and our sample is not fully representative of the general college student population in China. Second, we should ask what exact substance they are using so frequently. Take medicines as an example. What is the frequency of use of specific medication that participants are using? We do not know the frequency of use where participants answered benzodiazepines and/or selective serotonin reuptake inhibitors. Previous studies have revealed the past or current treatment, as psychotherapy and pharmacotherapy may have a significant impact on the clinical outcome explored in this study (substance use and affective symptoms): cognitive-behavioral therapy can be used to modify maladaptive automatic negative emotions of individuals about the pandemic (66); although benzodiazepines and selective serotonin reuptake inhibitor are known as the standard treatment of anxiety and insomnia, it is still a drug that may produce alterations in mood, cognitive, and psychomotor functions (67); the increase in benzodiazepines during the pandemic may be considered a proxy for increased anxiety that influences mood problems (68). The future study needs to examine the high-risk population with a current or history of pharmacotherapy and psychotherapy. There is a reason to suspect that social desirability could cause individuals to report inaccurate levels of participation in each of the mentioned behaviors, and the data here failed to capture this possibility. The last limitation is that we do not continue to track the emotional and substance problems of these CSAs after returning to the college campus. Further prospective cohort studies are needed to explore how Chinese college students' differences in mental health and substance use change under the COVID-19 influence more specifically to track the emotional and substance problems of CSAs back to college life and add items related to psychotherapy and pharmacotherapy to find more clinical implications. The future study could also find more social and biological mechanisms among these Chinese college students correlated to their mental health and substance use problems change.

## Data Availability Statement

The raw data supporting the conclusions of this article will be made available by the authors, without undue reservation.

## Ethics Statement

The studies involving human participants were reviewed and approved by Zhejiang Provincial People's Hospital. The patients/participants provided their written informed consent to participate in this study.

## Author Contributions

ML and CQ designed the study, managed the literature review, and drafted the report. ML, YW, and WS collected the data. ML and WS undertook statistical analyses and took the lead in writing the manuscript and approved the final manuscript. CQ commented on the manuscript. All authors contributed to the article and approved the submitted version.

## Conflict of Interest

The authors declare that the research was conducted in the absence of any commercial or financial relationships that could be construed as a potential conflict of interest.

## Publisher's Note

All claims expressed in this article are solely those of the authors and do not necessarily represent those of their affiliated organizations, or those of the publisher, the editors and the reviewers. Any product that may be evaluated in this article, or claim that may be made by its manufacturer, is not guaranteed or endorsed by the publisher.

## References

[1] Xiang Y.-T. Yang Y Li W Zhang L Zhang Q. Timely mental health care for the 2019 novel coronavirus outbreak is urgently needed. Lancet Psychiatry. (2020) 7:228–9. 10.1016/S2215-0366(20)30046-8 32032543 PMC7128153

[2] Duan L Shao X Wang Y Huang Y Miao J Yang X. An investigation of mental 399 health status of children and adolescents in china during the outbreak of COVID-19. J Affect Disord. (2020) 275:112–8. 10.1016/j.jad.2020.06.029 32658812 PMC7329661

[3] Brailovskaia J Cosci F Mansueto G Miragall M Herrero R Baños RM. The association between depression symptoms, psychological burden caused by covid-19 and physical activity: An investigation in Germany, Italy, Russia, and Spain. Psychiatry Res. (2021) 295:113596. 10.1016/j.psychres.2020.113596 33261924 PMC7688416

[4] Lai J Ma S Wang Y Cai Z Hu J Wei N. Factors associated with mental health outcomes among health care workers exposed to coronavirus disease 2019. JAMA Netw Open. (2020) 3:e203976–e203976. 10.1001/jamanetworkopen.2020.3976 32202646 PMC7090843

[5] Li M Su H Liao Z Qiu Y Chen Y Zhu J. Gender differences in mental health disorder and substance abuse of Chinese International College students during the COVID-19 pandemic. Front Psychiatry. (2021) 12:878. 10.3389/fpsyt.2021.710878 34484003 PMC8415825

[6] Zhang J Lu H Zeng H Zhang S Du Q Jiang T. The differential psychological distress of populations affected by the COVID-19 pandemic. Brain Behav Immunity. (2020) 87:49–50. 10.1016/j.bbi.2020.04.031 32304883 PMC7156946

[7] World Health Organization. Mental Health and Psychological Resilience During the COVID-19 Pandemic. (2020). Available online at: https://www.euro.who.int/en/health-topics/health-emergencies/ (accessed March 27, 2020).

[8] Liu Y Zhang N Bao G Huang Y Ji B Wu Y. Predictors of depressive symptoms in college students: a systematic review and meta-analysis of Cohort studies. J Affect Disord. (2019) 244:196–208. 10.1016/j.jad.2018.10.084 30352363

[9] Cao W Fang Z Hou G Han M Xu X Dong J. The psychological impact of the COVID-19 epidemic on college students in China. Psychiatry Res. (2020) 287:112934. 10.1016/j.psychres.2020.112934 32229390 PMC7102633

[10] Lei XY Xiao LM Liu YN Li YM. Prevalence of depression among Chinese University students: a meta-analysis. PLoS ONE. (2016) 11:e0153454. 10.1371/journal.pone.0153454 27070790 PMC4829172

[11] Wang C Zhao H. The impact of covid-19 on anxiety in chinese university students. Front Psychol. (2020) 11:1168. 10.3389/fpsyg.2020.01168 32574244 PMC7259378

[12] Son C Hegde S Smith A Wang X Sasangohar F. Effects of COVID-19 on college students' mental health in the United States: Interview survey study. J Med Internet Res. (2020) 22:e21279. 10.2196/21279 32805704 PMC7473764

[13] Patsali ME Mousa DP Papadopoulou EV Papadopoulou KK Kaparounaki CK Diakogiannis I. University students' changes in mental health status and determinants of behavior during the COVID-19 lockdown in Greece. Psychiatry Res. (2020) 292:113298. 10.1016/j.psychres.2020.113298 32717710 PMC7357537

[14] Conrad RC Koire A Pinder-Amaker S Liu CH. College student mental health risks during the COVID-19 pandemic: implications of campus relocation. J Psychiatr Res. (2021) 136:117–26. 10.1016/j.jpsychires.2021.01.054 33588225 PMC8635290

[15] Hahm HC Xavier Hall CD Garcia KT Cavallino A Ha Y Cozier YC. Experiences of covid-19-related anti-Asian discrimination and affective reactions in a multiple race sample of U.S. young adults. BMC Public Health. (2021) 21:1–11. 10.1186/s12889-021-11559-1 34407792 PMC8371291

[16] Forgey Q. Trump on 'Chinese virus' label: 'it's not racist at all'. POLITICO (2020). Available online at: https://www.politico.com/news/2020/03/18/trump-pandemic-drumbeat-coronavirus-135392 (accessed January 30, 2022).

[17] Haft SL Zhou Q. An outbreak of xenophobia: perceived discrimination and anxiety in Chinese American college students before and during the COVID−19 pandemic. Int J Psychol. (2021) 56:522–31. 10.1002/ijop.12740 33426695 PMC7962181

[18] Walters ST Roudsari BS Vader AM Harris TR. Correlates of protective behavior utilization among heavy-drinking college students. Addict Behav. (2007) 32:2633–44. 10.1016/j.addbeh.2007.06.022 17669596 PMC2001169

[19] Hao W Chen H Su Z. China: alcohol today. Addiction. (2005) 100:737–41. 10.1111/j.1360-0443.2005.01036.x 15918802

[20] Cui Y Ying M Fan H. Cigarette smoking practice and attitudes, and PROPOSED effective smoking cessation measures among college student smokers in China. Health Educ. (2012) 112:365–79. 10.1108/09654281211237180

[21] Mao R Li X Stanton B Wang J Hong Y Zhang H. Psychosocial correlates of cigarette smoking among college students in China. Health Educ Res. (2008) 24:105–18. 10.1093/her/cyn002 18281711

[22] Tam CC Benotsch EG Wang X Lin D Du H Chi P. Non-medical use of prescription drugs and cultural orientation among college students in China. Drug Alcohol Depend. (2018) 192:271–6. 10.1016/j.drugalcdep.2018.08.012 30300801

[23] Norton TR Lazev AB Sullivan MKJ. The “BUZZ” on Caffeine: Patterns of caffeine use in a convenience sample of college students. J Caffeine Res. (2011) 1:35–40. 10.1089/jcr.2010.0003

[24] Mahoney CR Giles GE Marriott BP Judelson DA Glickman EL Geiselman PJ. Intake of caffeine from all sources and reasons for use by college students. Clin Nutr. (2019) 38:668–75. 10.1016/j.clnu.2018.04.004 29680166

[25] Gaiha SM Cheng J Halpern-Felsher B. Association between youth smoking, electronic cigarette use, and COVID-19. J Adolesc Health. (2020) 67:519–23. 10.1016/j.jadohealth.2020.07.002 32798097 PMC7417895

[26] Graupensperger S Fleming CB Jaffe AE Rhew IC Patrick ME Lee CM. Changes in young adults' alcohol and marijuana use, norms, and motives from before to during the COVID-19 pandemic. J Adolesc Health. (2021) 68:658–65. 10.1016/j.jadohealth.2021.01.008 33781471 PMC8345007

[27] White HR Stevens AK Hayes K Jackson KM. Changes in alcohol consumption among college students due to covid-19: effects of campus closure and residential change. J Stud Alcohol Drugs. (2020) 81:725–30. 10.15288/jsad.2020.81.725 33308400 PMC7754852

[28] Kroenke K Spitzer RL Williams JB. The PHQ-9: validity of a brief depression severity measure. J Gen Int Med. (2001) 16:606–13. 10.1046/j.1525-1497.2001.016009606.x 11556941 PMC1495268

[29] Löwe B Decker O Müller S Brähler E Schellberg D Herzog W. Validation and standardization of the generalized anxiety disorder screener (GAD-7) in the general population. Med Care. (2008) 46:266–74. 10.1097/MLR.0b013e318160d093 18388841

[30] Luo W Zhong BL Chiu HF. Prevalence of depressive symptoms among Chinese university students amid the COVID-19 pandemic: a systematic review and meta-analysis. Epidemiol Psychiatr Sci. (2021) 30:1–21. 10.1017/S2045796021000202 33766163 PMC8047400

[31] Feng Y Xinyi X Aiai F. “yearning for it” and “No turning back”—a study on Chinese International High School students' motivations in the context of the COVID-19 outbreak. Int J Chin Educ. (2021) 10:221258682110466. 10.1177/22125868211046647

[32] Okruszek Ł Aniszewska-Stańczuk A Piejka A Wiśniewska M Zurek K. Safe but lonely? Loneliness, anxiety, and depression symptoms and COVID-19. Front Psychol. (2020) 11:579181. 10.3389/fpsyg.2020.579181 33343454 PMC7747668

[33] Hager NM Judah MR Milam AL. Loneliness and depression in college students during the COVID-19 pandemic: boredom and repetitive negative thinking as mediators (2020). Available online at: https://www.researchsquare.com/article/rs-101533/v1 (accessed November 3, 2020). 10.1007/s41811-022-00135-z PMC8990489 35432692

[34] Zhai Y Du X. Addressing collegiate mental health amid COVID-19 pandemic. Psychiatry Res. (2020) 288:113003. 10.1016/j.psychres.2020.113003 32315885 PMC7162776

[35] Wang Y Lu H Hu M Wu S Chen J Wang L. Alcohol consumption in China before and during COVID-19: preliminary results from an online retrospective survey. Front Psychiatry. (2020) 11:597826. 10.3389/fpsyt.2020.597826 33324263 PMC7723925

[36] Newman I Ding L Feng Y. Estimate of undergraduate university student alcohol use in China: a systematic review and meta-analysis. Arch Public Health. (2017) 75:1–13. 10.1186/s13690-017-0220-x 29238573 PMC5724242

[37] Kremer M Levy D. Peer effects and alcohol use among college students. J Econ Perspect. (2003) 22:189–206. 10.3386/w9876

[38] Wasserman D. Alcohol, other psychoactive substance use disorders, and suicide. Suicide. (2016) 73−84. 10.1093/med/9780198717393.003.000723192685

[39] Kotera Y Ozaki A Miyatake H Tsunetoshi C Nishikawa Y Tanimoto T. Mental health of medical workers in Japan during COVID-19: relationships with loneliness, hope and self-compassion. Curr Psychol. (2021) 40:6271–4. 10.1007/s12144-021-01514-z 33642837 PMC7896170

[40] Mansueto G Lopes FL Grassi L Cosci F. Impact of COVID-19 outbreak on Italian healthcare workers versus general population: results from an online survey. Clin Psychol Psychother. (2021) 28:1334–45. 10.1002/cpp.2644 34255890 PMC8426916

[41] Wartberg L Brunner R Kriston L Durkee T Parzer P Fischer-Waldschmidt G. Psychopathological factors associated with problematic alcohol and problematic internet use in a sample of adolescents in Germany. Psychiatry Res. (2016) 240:272–7. 10.1016/j.psychres.2016.04.057 27138817

[42] Branum AM Rossen LM Schoendorf KC. Trends in caffeine intake among US children and adolescents. Pediatrics. (2014) 133:386–93. 10.1542/peds.2013-2877 24515508 PMC4736736

[43] Malinauskas BM Aeby VG Overton RF Carpenter-Aeby T Barber-Heidal K. A survey of energy drink consumption patterns among college students. Nutr J. (2007) 6:1–7. 10.1186/1475-2891-6-35 17974021 PMC2206048

[44] Fulgoni VL Keast DR Lieberman HR. Trends in intake and sources of caffeine in the diets of US adults: 2001–2010. Am J Clin Nutr. (2015) 101:1081–7. 10.3945/ajcn.113.080077 25832334

[45] Drewnowski A Rehm C. Sources of caffeine in diets of US children and adults: trends by beverage type and purchase location. Nutrients. (2016) 8:154. 10.3390/nu8030154 26978391 PMC4808882

[46] Bergstrom L. The use of multiple dietary supplements. J Diet Suppl. (2009) 6:1–8. 10.1080/19390210802687262 22435348

[47] Margaritis I Houdart S El Ouadrhiri Y Bigard X Vuillemin A Duch é P. How to deal with COVID-19 epidemic-related lockdown physical inactivity and sedentary increase in youth? Adaptation of Anses' benchmarks. Arch Public Health. (2020) 78:1–6. 10.1186/s13690-020-00432-z 32514348 PMC7267755

[48] Marku M McCarthy BC. Dietary supplement use, knowledge, and perceptions among student pharmacists. Am J Pharm Educ. (2017) 81:6775. 10.5688/ajpe6775 29367781 PMC5774201

[49] Sirico F Miressi S Castaldo C Spera R Montagnani S Di Meglio F. Habits and beliefs related to food supplements: results of a survey among Italian students of different education fields and levels. PLoS ONE. (2018) 3:e0191424. 10.1371/journal.pone.0191424 29351568 PMC5774790

[50] Liu H Zhang S Zou H Pan Y Yang Q Ouyang Y. Dietary supplement use among chinese primary school students: a cross-sectional study in Hunan Province. Int J Environ Res Public Health. (2019) 16:374. 10.3390/ijerph16030374 30699949 PMC6388182

[51] Silva-Jones J. Why Can't We Sleep? Impact of Race and Social Status on Sleep in College (Master's Theses, Dissertations). Graduate Research and Major Papers Overview (2019).

[52] Cifre A Budnick C Walters K. College student sleep disturbances and psychosocial functional impairment. Sleep Med. (2019) 64:S75. 10.1016/j.sleep.2019.11.208

[53] Friedrich A Schlarb AA. Let's talk about sleep: a systematic review of psychological interventions to improve sleep in college students. J Sleep Res. (2017) 27:4–22. 10.1111/jsr.12568 28618185

[54] Wu Y Fan H Guo Z Wei L. Factors associated with smoking intentions among Chinese college students. Am J Mens Health. (2018) 13:155798831881828. 10.1177/1557988318818285 30813857 PMC6775548

[55] Daniel C Haddad C McConaha JL Lunney P. Electronic cigarettes: their role in the lives of college students. J Pharm Pract. (2021). 10.1177/08971900211026841. [Epub ahead of print] 34155945

[56] Choi D Ota S Watanuki S. Does cigarette smoking relieve stress? Evidence from the event-related potential (ERP). Int J Psychophysiol. (2015) 98:470–6. 10.1016/j.ijpsycho.2015.10.005 26497442

[57] Luzius A Dobbs PD Jozkowski KN. College students' reasons for using different e-cigarette products: a mixed methods analysis. J Am Coll Health. (2019) 68:832–8. 10.1080/07448481.2019.1618313 31157606

[58] Cohn A Villanti A Richardson A Rath JM Williams V Stanton C. The association between alcohol, marijuana use, and new and emerging tobacco products in a young adult population. Addict Behav. (2015) 48:79–88. 10.1016/j.addbeh.2015.02.005 26042613

[59] Ramo DE Liu H Prochaska JJ. Tobacco and marijuana use among adolescents and young adults: a systematic review of their co-use. Clin Psychol Rev. (2012) 32:105–21. 10.1016/j.cpr.2011.12.002 22245559 PMC3267894

[60] Arria AM Caldeira KM Vincent KB O'Grady KE Wish ED. Perceived harmfulness predicts nonmedical use of prescription drugs among college students: interactions with sensation-seeking. Prev Sci. (2008) 9:191–201. 10.1007/s11121-008-0095-8 18633709 PMC2574828

[61] Sullivan L Winkelman J. Sleep and marijuana products in 2020. Curr Sleep Med Rep. (2020) 6:208–11. 10.1007/s40675-020-00187-7

[62] Goodhines PA Gellis LA Park A. 0194 alcohol and marijuana use for sleep aid in college students: a daily diary investigation. Sleep. (2018) 41(suppl_1):A76. 10.1093/sleep/zsy061.193

[63] Lee CM Cadigan JM Rhew IC. Increases in loneliness among young adults during the COVID-19 pandemic and association with increases in mental health problems. J Adolesc Health. (2020) 67:714–7. 10.1016/j.jadohealth.2020.08.009 33099414 PMC7576375

[64] Levine MP. Loneliness and eating disorders. J Psychol. (2012) 146:243–57. 10.1080/00223980.2011.606435 22303623

[65] Stickley A Koyanagi A. Loneliness, common mental disorders and suicidal behavior: findings from a general population survey. J Affect Disord. (2016) 197:81–7. 10.1016/j.jad.2016.02.054 26971125

[66] Swartz HA. The role of psychotherapy during the COVID-19 pandemic. Am J Psychother. (2020) 73:41–2. 10.1176/appi.psychotherapy.20200015 32516084

[67] Cosci F Mansueto G Faccini M Casari R Lugoboni F. Socio-demographic and clinical characteristics of benzodiazepine long-term users: results from a tertiary care center. Compr Psychiatry. (2016) 69:211–5. 10.1016/j.comppsych.2016.06.008 27423363

[68] de Dios C Fernandes BS Whalen K Bandewar S Suchting R Weaver MF. Prescription fill patterns for benzodiazepine and opioid drugs during the COVID-19 pandemic in the United States. Drug Alcohol Depend. (2021) 229:109176. 10.1016/j.drugalcdep.2021.109176 34808468 PMC8595244

